# Supplementary material for: Unraveling the dynamic transcriptomic changes during the dimorphic transition of Talaromyces marneffei through time-course analysis
Source: Front Microbiol. 2024 Apr 24;15:1369349. doi: 10.3389/fmicb.2024.1369349 (PMC11076716; doi:10.3389/fmicb.2024.1369349)
Supplement: Supplementary file 1 [file Data_Sheet_1.docx]

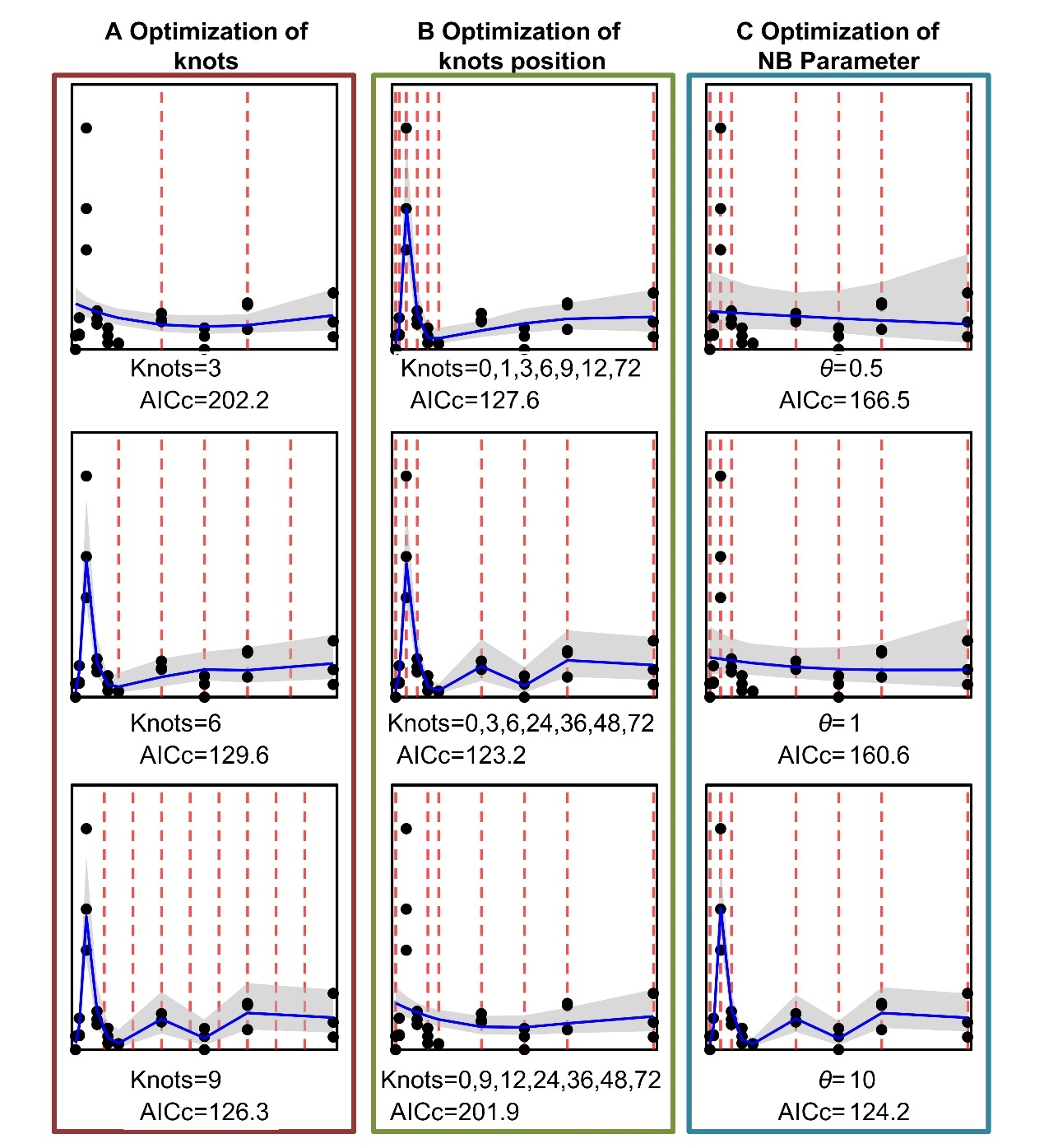


**Figure S1. Gene-specific optimization of DyGAM-NS model. (A)** Refining the DyGAM-NS model for gene-specific NS partition numbers. AICc values depict the model’s fit to segmented average gene expression, partitioned into 3, 6, and 9 sections. **(B)** Fine-tuning knots position in the model. AICc values showcase the impact of different knots position with a constant number of knots. **(C)** Tailoring the model for gene expression distribution. AICc values reveal the model’s performance under varied negative binomial distribution dispersion coefficients of 0.5, 1, and 10 for gene expression.

**
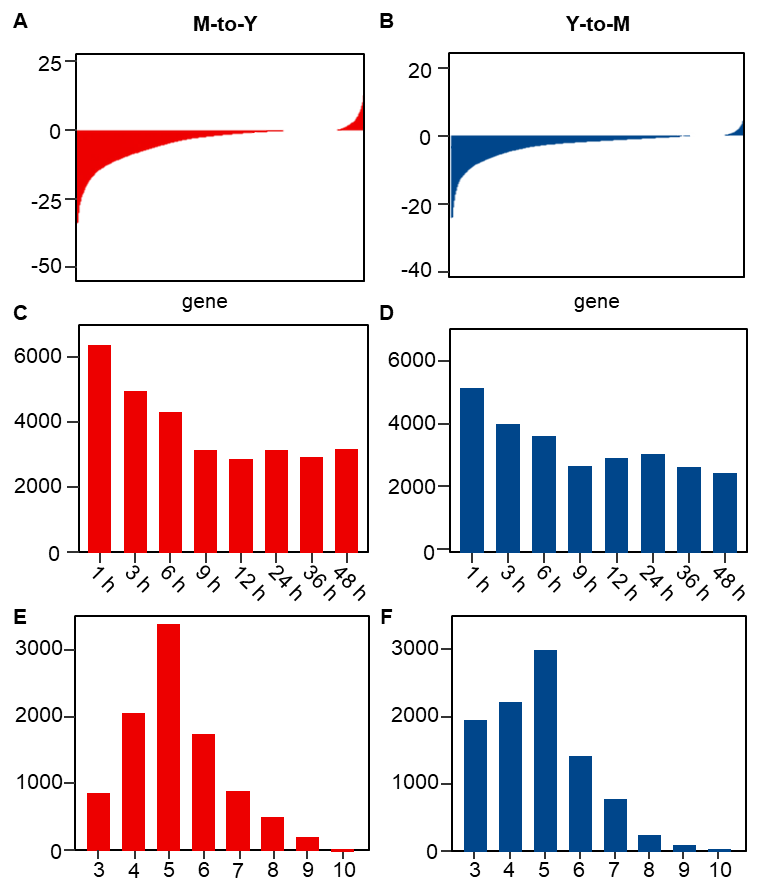
**

**Figure S2. Performance of DyGAM-NS model after parameter optimization. (A)** and **(B)** AICc delta pre- and post-optimization. The y-axis showcases the difference in AICc values post-optimization compared to pre-optimization (ΔAICc = AICc post-optimization - AICc pre-optimization), and the x-axis arranges genes based on AICc differences. **(C)** and **(D)** Optimal knots position. The x-axis showcases the preferred knots position, with the y-axis indicating the gene count. **(E)** and **(F)** Optimal numbers of knots. The x-axis illustrates the ideal number of knots for each gene, while the y-axis displays the gene count.

**
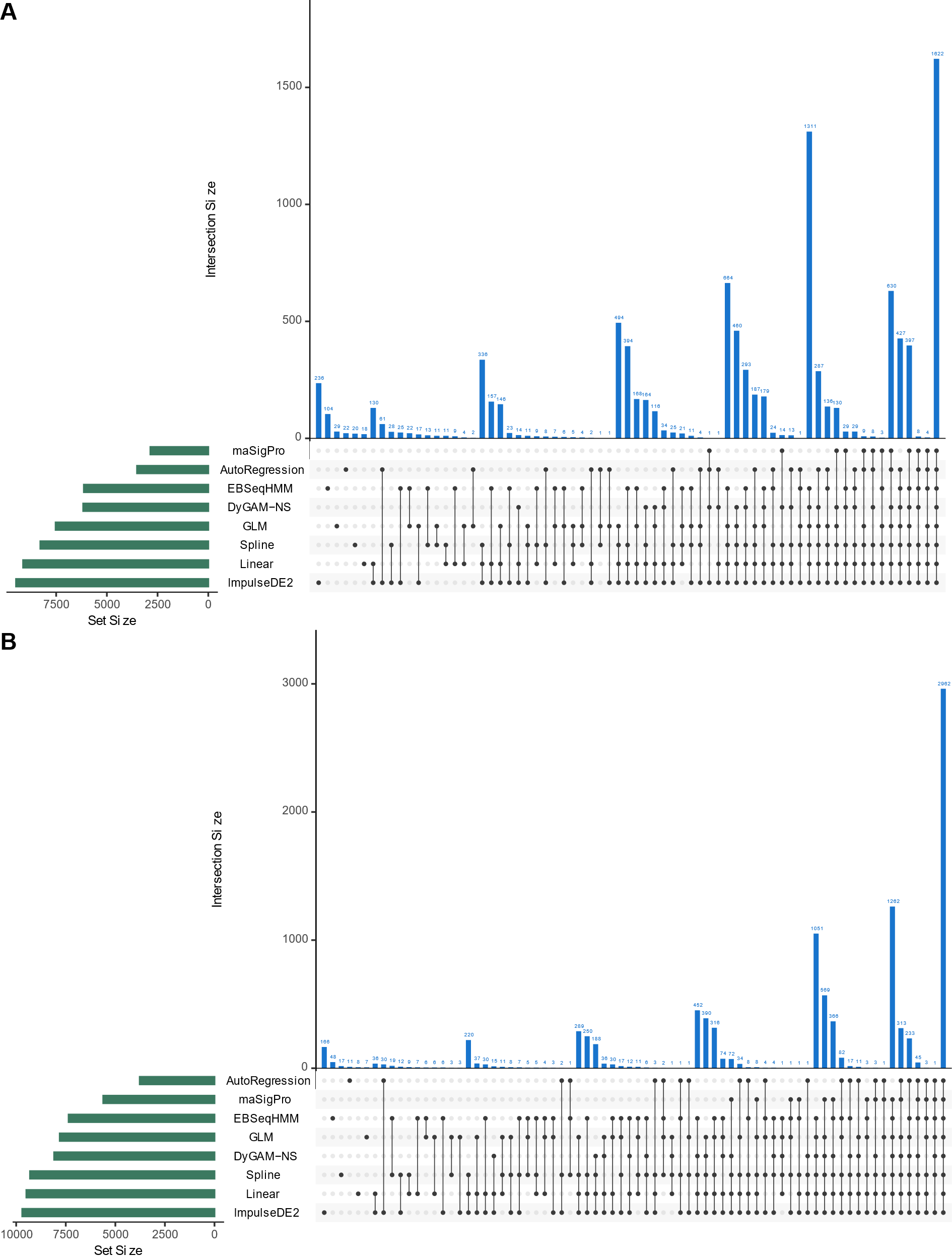
**

**Figure S3. Comparison of time-course analysis methods.** Comparison of genes inducing M-to-Y transition **(A)** and Y-to-M transition **(B)**.


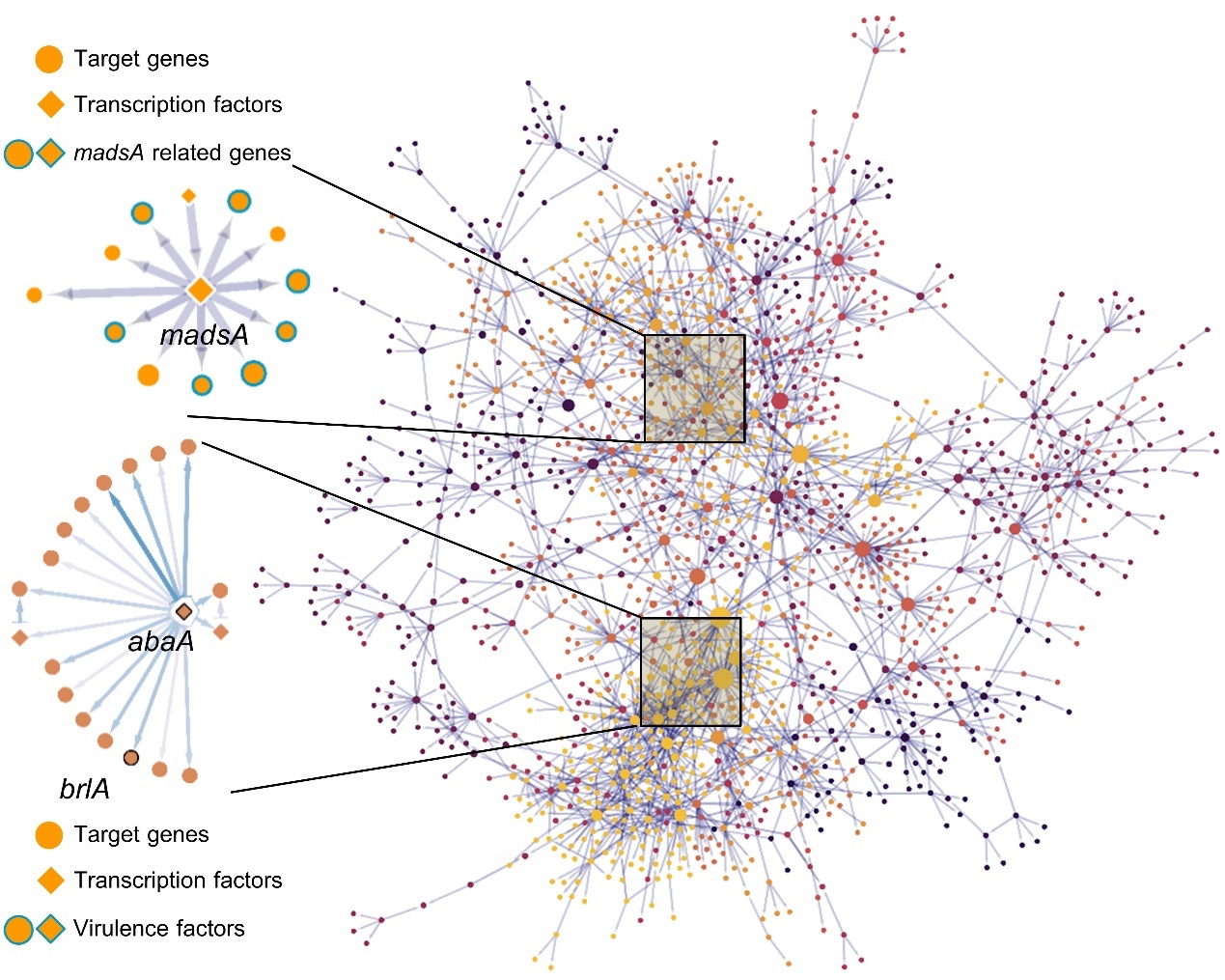

**Figure S4. Gene regulatory network of dimorphic transition of *T. marneffei*.** Visualization of gene regulatory network of dimorphic transition. Each point represents a gene, the size of points represents importance of genes in the network, which is measured with betweenness, and the color of points indicates the module to which the corresponding gene belongs. The light grey lines represent strong regulatory relationship between corresponding gene pairs. In the upper left, the figure illustrates the correlation between *madsA* and its downstream regulatory genes. Below, the relationship between *abaA* and its downstream regulatory genes is depicted. Circular nodes without borders denote target genes, diamond nodes without borders represent transcription factors, and bordered nodes represent virulence factors validated in prior studies on *T. marneffei*.
